# The Correlation between Head of Bed Angle and Intra-Abdominal Pressure of Intubated Patients; a Pre-Post Clinical Trial

**DOI:** 10.22037/aaem.v9i1.1065

**Published:** 2021-03-06

**Authors:** Sedigheh Samimian, Sadra Ashrafi, Tahereh Khaleghdoost Mohammadi, Mohammad Reza Yeganeh, Ali Ashraf, Hamideh Hakimi, Maryam Dehghani

**Affiliations:** 1 Clinical Research Development Unit of Poursina Hospital, Guilan University of Medical Sciences, Rasht, Iran.; 2 Student Research Committee, Chronic Kidney Disease Research Center(CKDRC), Shahid Beheshti University of Medical Sciences, Tehran, Iran.; 3 Department of Medical-Surgical Nursing, Shahid Beheshti Faculty of Nursing and Midwifery, Guilan University of Medical Sciences, Rasht, Iran.; 4 Department of Nursing, Lahijan Branch, Islamic Azad University, Lahijan, Iran.; 5 Nahavand School of Allied Medical Sciences, Hamadan University of Medical Sciences, Hamadan, Iran.

**Keywords:** Pressure, Intra-abdominal Hypertension, Head of Bed, Critical care, Compartment syndrome, Supine Position

## Abstract

**Introduction:**

The recommended position for measuring Intra-Abdominal Pressure (IAP) is the supine position. However, patients put in this position are prone to Ventilator-associated pneumonia. This study was done to evaluate the relationship between bed head angle and IAP measurements of intubated patients in the intensive care unit.

**Methods:**

In this clinical trial, seventy-six critically ill patients under mechanical ventilation were enrolled. IAP measurement was performed every 8 hours for 24 hours using the KORN method in three different degrees of the head of bed (HOB) elevation (0°, 15°, and 30°). Bland-Altman analysis was performed to identify the bias and limits of agreement among the three HOBs. According to World Society of the Abdominal Compartment Syndrome (WSACS), we can consider two IAP techniques equivalent if a bias of <1 mmHg and limits of agreement of - 4 to +4 were found between them. Data were analyzed using SPSS statistical software (v. 19), and the significance level was considered as 0.05.

**Results:**

The prevalence of intra-abdominal hypertension was 18.42%. Mean ± standard deviation (SD) of IAP were 8.44 ± 4.02 mmHg for HOB angle 0°, 9.58 ± 4.52 for HOB angle 15°, and 11.10 ± 4.73 for HOB angle 30^o^ (p = 0.0001). The IAP measurement bias between HOB angle 0°and HOB angle 15° was 1.13 mmHg. This bias was 2.66 mmHg between HOB angle 0° and HOB angle 30°.

**Conclusion:**

Elevation of HOB angle from 0 to 30 degree significantly increases IAP. It seems that the measurement of IAP at HOB angle 15° was more reliable than 30°.

## Introduction

Intra-abdominal pressure (IAP) is increasingly considered an important pathological factor among the patients admitted to intensive care unit (ICU) (1). Elevated IAP is a frequent cause of morbidity and mortality among ICU patients (2). Recurrent and pathological elevation of IAP (≥12 mmHg) without organ dysfunction is called Intra-abdominal Hypertension (IAH), and if it is associated with organ dysfunction, abdominal compartment syndrome (ACS) occurs (3, 4)

The prevalence of IAH in intensive care patients has been reported as 18% to 58.8% (5, 6). This wide range of prevalence is due to the differences in clinical settings (surgical or medical) and conditions (trauma, burn and postoperative patients), the method chosen for IAP measurement, and also the lower limit selected for IAH definition (7). The clinical development of IAH is a silent process and it is therefore, not diagnosed until its progress has become complete (8). The sensitivity of clinical examination in diagnosis of IAH is only 40% to 60%, so IAP should be measured periodically not to miss IAH/ACS diagnosis (2, 8). According to the recommendation of the World Society of Abdominal Compartment Syndrome (WSACS), IAP should be routinely measured in patients with at least two IAH risk factors (9). There are various methods to measure IAP. The measurement of the intra-vesical pressure is recognized as the standard method of measuring IAP according to WSACS (10, 11). The standard position for measuring IAP is the supine position or zero degrees of the head of bed (HOB). However, for the patients admitted to ICU, this position is not desired, and it leads to unavoidable outcomes including ventilator-associated pneumonia (VAP), respiratory distress, hemodynamic changes and so on, particularly when IAP is continuously measured (2, 10, 12, 13).

Putting the patient in supine position is contrary to the policies recommended by Centers for Disease Control (CDC). These policies prevent aspiration pneumonia and ventilator-associated pneumonia. On the other hand, measurement of IAP at 0° of HOB, in case of patients' intolerance and the existence of both abdominal and bladder muscle retraction, leads to the false increase of IAP (1, 14, 15) or IAP measurements at 0° of HOB may underestimate or overestimate the IAP that the patient is experiencing during the intervals of measurements (16, 17).

There is some evidence that body position can affect measured IAP values, but the effect of HOB elevation degree, which is applied for changing the position of ICU patients, is not clearly defined (18). This study was done in order to evaluate the relationship between bed head angle and IAP measurements of intubated patients in the intensive care unit.

## Methods

**Study Design and settings**

The current study is a clinical trial aimed to compare the changes of IAP with HOB angle at 0°, 15°and 30° among the patients admitted to ICU. This study was conducted on 76 patients admitted to general ICU, neurology ICU and trauma wards of educational hospitals of Rasht, Iran, during 3 months. Prior to the initiation of the study, approvals from the Ethics Committee of Guilan University of Medical Sciences (Ethics code: 290963812) and Iranian Registry of Clinical Trials (Clinical trial number: IRCT201010214787N2) were secured. Out of 289 patients evaluated in terms of inclusion criteria, the patients who met the criteria were enrolled in the study after obtaining consent from their legal guardian.

**Participants**

Patients admitted to ICU during the study period with age over 18 years, Richmond Agitation Sedation scale (RASS) score equal to -4 or -5, mechanically ventilated for at least 24 hours, lacking spinal cord damage, with normal Intracranial Pressure, without recent bladder surgery, and without both nasogastric tube and Foley catheter, were included. Exclusion criterion was intolerance to HOB elevation, which didn’t occur during our study. 

**Data gathering**

The research instrument was obtained from WSACS and consisted of three sections: The first section was dedicated to the personal characteristics of the patients, including age, gender, body mass index, disease diagnosis and the length of stay. The second section included Sequential Organ Failure Assessment (SOFA) score, and the third section was related to IAP measurement and recording, ventilator mode variables, mean arterial pressure (MAP), mean IAP, mean airway pressure, maximum airway pressure, plateau pressure, abdominal perfusion pressure (APP), and positive end-expiratory pressure measurement at 0°, 15° and 30° head positions. SOFA scores were calculated based upon the 24-hour period prior to the first IAP measurement.

**Intervention**

IAP measurement was performed through KORN method using Foley catheter (4, 13). After clamping the tube at the end of the collection bag, under sterile conditions, the aspiration port was connected to a short 18-G catheter with three stopcocks. They were connected to an intravenous infusion set, a syringe for flushing and draining the tube system, and a water manometer. Zero reference was at the iliac crest in the mid-axillary line. Twenty-five milliliters of saline were instilled into the bladder. Three sets of IAP measurement were done with HOB angle elevated at 0°, 15°, and 30^o^ every 8 hours during 24 hours (8^AM^, 4^PM^, 12^MN^). The patients were followed during this period. All patients were flexed at the waist without readjustment of the manometer's zero reference. IAPs were measured at end-expiration with a minimum of 1 minute delay after patient positioning, for equilibration. IAPs were measured in cmH2O and then converted into mmHg through multiplying by 0.74. The measurement of the bed angle was carried out by the index in the rail beside the bed based on the horizon level in each condition. To avoid technical errors, the same person carried out all of the measurements. After each measurement, the clamp was opened to discharge the normal saline from the patient’s bladder completely. Also, the patient's nurse was noted to subtracts the volume of the normal saline infused into the bladder from the total volume of urine drainage of the patient in that hour. By monitoring the patient’s ventilator, other pressure variables (like mean airway pressure, maximum airway pressure, etc.) were recorded. Importantly, the duration of each measurement was about 7 to 8 minutes. The triplicate IAP measurements for each body position were done. The IAP was measured 228 times in each HOB angle and 684 times in total. During the measurement, in the case of IAH, the nurse or physician was called for treatment.

In the present study, IAP was an independent variable. IAH (IAP≥12 mmHg) was categorized as indicated below: grade I (12-15 mmHg), grade II (16-20 mmHg), grade III (21-25 mmHg), grade IV (>25 mmHg) (2).

**Statistical Analysis**

The data were collected, encoded, and analyzed via descriptive and inferential statistics using the SPSS statistical software (v. 19). Mean ± standard deviation (SD) was calculated for quantitative variables and frequency (%) for qualitative variables. Kolmogorov Smirnov test was utilized to check the normal distribution of data. All data were distributed normally. Repeated measure analysis of variance (ANOVA) and Bonferroni post hoc tests were used to compare the mean abdominal pressure at different measurement angles. Considering the insignificance of the Mauchly test, Sphericity and repeated measures ANOVA tests were used to compare the trend and amount of IAP changes with qualitative variables. To investigate the correlation of quantitative variables with mean IAP, Pearson test and Fisher’s exact test were used. Bland-Altman analysis was performed to identify the bias and limits of agreement among the three positions. The WSACS states that a bias of <1 mm Hg and limits of agreement between -4 and +4 are required for considering the two IAP techniques equivalent (1, 19). P-value of <0.05 was considered statistically significant. 

## Results

**Figure 1:**
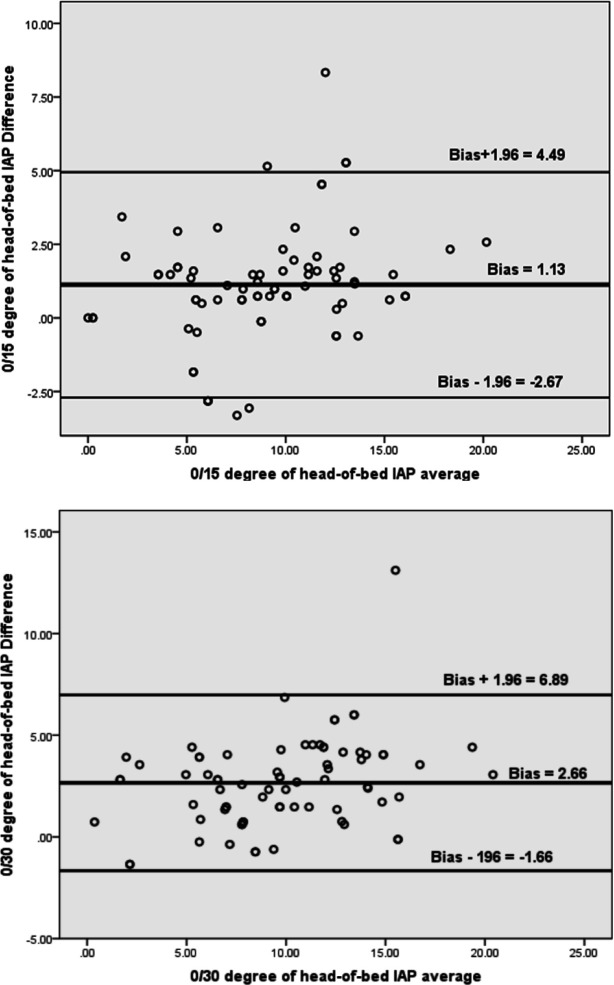
The bias and agreement limit between intra-abdominal pressures (IAPs) measured at 0° and 15° and 0° and 30° head of bed elevation

289 patients were evaluated, out of which 76 cases with the mean age of 50.31 ± 20.47 years met the predefined inclusion criteria and were enrolled in the study (72.4% male). The baseline characteristics of patients are presented in table 1. The IAP was measured 228 times in each HOB angle and 684 times in total. The prevalence of IAH in the current study was 18.42%, and there was no case of abdominal compartment syndrome. The results showed that mean IAP was 8.44 ± 4.02 mmHg in 0°, 9.58 ± 4.52 mmHg in 15°, and 11.10 ± 4.73 mmHg in 30° of HOB (p < 0.001) (Table 2). Table 3 shows the prevalence of IAH in three different HOB degrees, 0°, 15° and 30°. Normal IAP prevalence was reduced from 0° (81.6%) to 15° (65.8%) and 30^°^ (57.9%), and grade III IAH prevalence was increased from 0° to 30^°^ (3.9%). In other words, with the increase in HOB angle, IAP was changed from a normal state to IAH (p = 0.04). Figure 1 shows the bias (1.13) and agreement limit (-2.67 to 4.94) between IAPs measured at 0° and 15°. Figure 1 also indicates bias and agreement limit between IAPs measured at 0° and 30° (The bias was 2.66 and the agreement limit was -1.66 to 6.89).

## Discussion

The results showed that increase in HOB angle from 0 to 30 degrees led to significant increase in IAP and the prevalence of IAH also increased from 0° to 30^o^. In other words, by increasing HOB angle, the normal IAP turned in to IAH. 

Khalaf Mahran et al. showed the significant mean IAP changes between different HOB elevations (HOB angle 0°, HOB angle 15°, and HOB angle 30°) (20). Vasquez et al. found that IAP changed from 10.2 mmHg at 0° to 12.4 mmHg at 15° and 14 mmHg at 30^° ^(18). In another study, McBeth et al. showed that with increase in HOB, IAP increased and this difference was significant when it changed from 30 degrees to 45 degrees (1). Unlike the mentioned studies, Cresswell et al. showed that the mean upper intra-abdominal pressure decreased by 2.1 mmHg when HOB increased by 30°. They explained that when the HOB was elevated, the wall of the abdominal muscles relaxed and the abdominal wall tension decreased (21). Another reason can be the pressure of organs on the abdomen due to the gravity force resulting from the change of body position (2).

Although measuring IAP at 0° can predispose the patients to pneumonia and some other side effects, the IAPs measured at 0°, 15° and 30° cannot be considered precisely equal. So, if a decision needs to be made for patients' treatment and management based on the measured IAP at HOB angles other than 0°, these differences should be noticed. 

The changes of IAP between 0°, 15°and 30° HOB angles had a significant correlation with the age and the differences between IAPs increase by age. Murcia-Saez et al. also found a significant correlation between the age and mean IAP in their study participants (P=0.001, R=0.36) (10). Ejike et al., in a study among the age group below 18 years old, found a significant correlation between the age and IAP (P = 0.02) (12). But the study by Vasquez et al. showed no correlation between the age and IAP (P=0.3) (18). 

It can be said that the prevalence of comorbidities is higher in older age and the comorbidities themselves can increase IAP. Therefore, the difference in the results of different studies, mentioned previously, may be due the differences in participants and measurement techniques. 

BMI was considered an effective factor in our study, and it was significantly associated with IAP (P=0.007). Vasquez et al., in their study, showed that there was a significant correlation between BMI and mean IAP. They found that BMI was in charge of 25-36% of the changes in IAP (18). Blaser et al. and McBeth et al. also found the correlation between these two variables to be significant (P=0.01) (1, 22). It seems that the fat tissue in the abdominal cavity (central obesity) increased IAP among people with high BMI by a direct effect on abdominal cavity and the bottom of pelvis (23). 

There was a significant correlation between the changes of IAP and disease diagnosis (P=0.04) as the change increased among non-trauma patients compared to trauma patients. In a study by McBeth et al., it was shown that IAP and the diagnosis of Neurologic disease had a significant correlation (P=0.001). In contrast, in surgical and trauma patients, no significant correlation was observed (1). In the epidemiological multi-centered study by Malbrain et al., there was no significant correlation between the diagnosis of medical and surgical diseases with IAP (24). Moreover, Ejik et al. also concluded that there was no significant correlation between disease diagnosis and IAP changes in patients below 18 years old (12). However, in another study, which was done by Murcia Saez et al., the mean IAP was high among surgical patients (P=0.001) (10). Based on the distribution of disease diagnosis in our study, the studied patients were divided into two groups of trauma and non-trauma patients. Various studies revealed different results about the correlation of IAP and disease diagnosis. This may be due to the difference in categories of disease diagnosis. Therefore, it seems that more studies are needed in the future in various groups of patients in terms of disease type.

There was a significant correlation between the IAPs at 0°, 15° and 30^o^ with various respiratory pressures, mean airway pressure, plateau pressure and positive end-expiratory pressure (P=0.05). The mean arterial pressure and IAP also significantly correlated with each other at the three angles (P=0.0001). The rib cage and abdominal cavity are connected via diaphragm as averagely, 50% (25 to 80%) of the pressure of these two sections (abdomen and ribcage) are transferred to each other. With increase in IAP, the diaphragm is elevated and leads to the compression of lungs and increase in the pressure of ribcage. Following the rise in the pressure of the ribcage, increase in mean airway pressure, maximum airway pressure, plateau pressure, and positive end-expiratory pressure are expected. With an increase in ribcage pressure, the heart function and mean arterial pressure are also reduced. Eventually, these events lead to the increase of IAP (25, 26).

Based on the comparison of the bias and agreement limit of the measured values between 0° and 15° (1.13, -2.67 to +4.94), and between 0° and 30° (2.66, -1.66 to 6.89) with bias and agreement limit (-4 to +4) suggested by the WSACS, it was shown that the bias at 15 degrees compared to 0° (standard position) was closer to the required bias. The lower limit of the agreement in both 15°and 30^o^ compared to 0° (standard position) were approximately equal. However, the upper limits of agreement in both angles were higher than the WSACS upper limit, and the limit was much more near to the WSACS limit at 15°. It was found that 87.6% of the agreement limit obtained between 0°and 15° in this study was in the range proposed by the WSACS, and only about 13% was out of this limit and about the limits obtained between 0° and 30°, 66.19% of the agreement limit obtained was in the range and 33.81% was out. In the study by Cheatham et al., it was shown that the bias between 0 and 15 degrees was 1.5 mmHg, and the agreement limit was -2.8 to 5.8 and the bias between 0 and 30 degrees was 3.7 mmHg ,and the agreement limit was -2.2 to 9.6 (2).

According to the results of previous studies, as also found in our study, an increase in HOB will lead to a clinically important increase in IAP. Moreover, it can be concluded that IAP at 15°, in comparison to 30°, can be considered more equal to IAP at standard position. So considering HOB seems to be important in interpretation of IAP.

The elevation of HOB significantly increased IAP. Although IAP needs to be measured for the patients admitted to ICU, putting the patient at zero degree is not possible in some conditions because it led to signs of intolerance to the position and unavoidable complications among the patients. The results of our study showed that 87.6% of IAPs measured at 15° were in the agreement limit in accordance with the WSACS. The HOB angle in ICUs is often recommended to be adjusted to 30 degrees. It seems that measuring IAP at 15° HOB angle is more reliable than 30° in ICU patients who cannot tolerate supine position. To determine the appropriate HOB angle to measure IAP in ICU patients, further studies are needed to reveal any other factors that may affects IAP, besides HOB. There is also the need to evaluate whether the increase in HOB leads to an equal increase in the IAPs of four quadrants of the abdomen cavity or not. As long as more extensive studies evaluating the effects of position on IAP aren’t available, it's better to measure IAP at 0° or standard position. 

**Table 1:** Baseline characteristics of studied population (n = 76)

**Variables**	**Values**
**Age (year)**	
Mean ± SD	50.31 ± 20.47
**BMI (kg/m** ^2^ **)**	
Mean ± SD	23.70 ± 6.94
**Gender**	
Male	55 (72.4)
Female	21 (27.6)
**Cause of admission**	
Trauma	46 (60.5)
Non-trauma	30 (39.47)
**Mechanical ventilation Modes**	
SIMV	68 (89.5)
BIPAP	2 (2.6)
CPAP	6 (7.9)
**Length of stay (day)**	
Mean ± SD	5.43 ± 5.17
**SOFA Score **	
Mean ± SD	6.85 ± 3.07
**Pressures**	
Mean arterial (mmHg)	95.15 ± 17.66
PIP (cmH_2_O)	26.59 ± 6.93
Plateau (cmH_2 _o)	17.05 ± 5.53
Mean air way (cmH_2 _o)	9.67 ± 2.42
APP (mmHg)	86.71 ± 1.65
IAP at HOB angle 0 (mmHg)	8.44 ± 4.02
IAP at HOB angle 15 (mmHg)	9.58 ± 4.52
IAP at HOB angle 30 (mmHg)	11.10 ± 4.73

Data presented as mean ± standard deviation (SD) or number (%), BMI= Body Mass Index, SIMV = Synchronized Intermittent Mandatory Ventilation, BIPAP= Bi-level positive airway pressure, CPAP= Continuous positive airway pressure, SOFA= Sequential Organ Failure Assessment, PIP= Peak inspiratory pressure, APP= Abdominal perfusion pressure, IAP= Intra-abdominal pressure, HOB: head of bed.

**Table 2:** Comparison of mean intra-abdominal pressure (IAP) at 0, 15 and 30 degrees of bed head angle and different measurement times

**Times** ^#^	**Head of bed angle (degree)**			**P value**			
	0°	15°	30°	0°/15°/30°	0°/15°	0°/30°	15°/30°
First	8.49± 4.27	9.61 ± 4.60	11.04 ±4.70	0.001	0.001	0.001	0.001
Second	8.26 ± 4.16	9.52 ± 4.44	11.10 ± 4.71	0.001	0.001	0.001	0.001
Third	8.57 ±4.14	9.60 ± 4.54	11.15 ± 4.79	0.001	0.001	0.001	0.001
*Mean	8.44 ± 4.02	9.58 ± 4.52	11.10 ±4.73	0.001	0.001	0.001	0.001

Data are presented as mean ± standard deviation; *Mean IAP in 24 hours; # times of measurement (the IAP was measured 3 times in each degree of head of bed angle).

**Table 3:** Intra-abdominal pressure changes at 0°, 15° and 30° of head of bed angle based on grades of Intra-abdominal hypertension

Angle^#^	Normal	Grade I	Grade II	Grade III	Grade IV	Total	P*
0°	62 (81.6)	12 (15.8)	2 (2.6)	0 (0)	0 (0)	76 (100)	0.04
15°	50 (65.8)	20 (26.3)	5 (6.6)	1 (1.3)	0 (0)	76 (100)	
30°	44 (57.9)	22 (28.9)	7 (9.2)	3 (3.9)	0 (0)	76 (100)	

Data are presented as number (%). * Fisher's exact test; # the angle of head bed elevation.

## Limitation

One of the limitations of our study was lack of a special kit for measuring the IAP. Also, the patients who would not endure an elevation in HOB were excluded in our study. Unfortunately, many of these patients were at high risk for IAH/ACS. One of the positive aspects in our study is the evaluation of a larger sample size. According to the WSACS website, the minimum sample size for research on the IAP measurement methods is 20 cases.

## Conclusion

Elevation of HOB angle from 0 to 30 degree significantly increases IAP. It seems that the measurement of IAP at HOB angle 15° was more reliable than 30°.
